# Functional enhancement of acute infracted heart by coinjection of autologous adipose-derived stem cells with matrigel

**DOI:** 10.55730/1300-0152.2653

**Published:** 2023-05-04

**Authors:** Bao-Zhu WANG, Meng-Meng WANG, Yan LI, Mei-Hua SHAO, Dan ZHANG, Shi-Qi YAN, Xiang MA, Yi-Tong MA

**Affiliations:** 1 Department of Cardiology of the First Affiliated Hospital, Xinjiang Medical University, Urumqi, P.R. China; 2 Department of Intensive Care Unit of the Fifth Affiliated Hospital, Xinjiang Medical University, Urumqi, P.R. China

**Keywords:** Matrigel, myocardial infarction, stem cells, transplantation, autologous

## Abstract

Recent clinical developments in tissue bioengineering have applications in acute cardiac ischemia and infarction and include the use of stem cells that combine injectable scaffold material. This study aimed to evaluate the effects of adipose-derived stem cells (ADSCs) that combine the Matrigel scaffold on cardiac morphology/functions. The autologous ADSCs myocardial infarction (MI) model was induced by the permanent ligation method of the left anterior descending coronary artery (LAD). MI-operated rats were randomly divided into PBS group, Matrigel group, PBS plus ADSCs group (PBS+ADSCs), and Matrigel plus ADSCs group (Matrigel+ADSCs). Matrigel was used as an injectable scaffold. Rats with a 1-week-old myocardial infarction were injected with 2 × 10^6^ labeled ADSCs in the border area of the ischemic heart. Heart function was determined by echocardiography. The hemodynamics, cardiac structure, and graft characteristics were evaluated. The ADSCs were successfully isolated and identified, demonstrating a good proliferative status and cell retention in the Matrigel. ADSCs+Matrigel exhibited the most improved heart functions (LVESD, LVEDD, LVFS, LVEF) compared to those of other groups (p < 0.05). ADSCs+Matrigel significantly reduced infarct size compared to other groups (p < 0.05). Cotransplantation of ADSCs and Matrigel showed the best effect on maintaining the thickness of the ventricular wall compared to the other groups (p < 0.05). Engrafted ADSCs played a role in the formation of the neovasculature in myocardial infarction. ADSCs+Matrigel triggered the greatest enhancement in arteriole density than other groups (p < 0.05). Cotransplanting with ADSCs and Matrigel showed significantly higher levels of cardiac troponin T (cTnT), NK2-transcription factor related locus-5 (Nkx2.5), von Willebrand factor (vWF) than the other groups (p < 0.05). In conclusion, this study demonstrated that cotransplanting ADSCs with Matrigel resulted in improved cardiac morphology and cardiac function in the rat model of myocardial infarction.

## 1. Introduction

Myocardial infarction (MI) can cause cardiomyocyte death, scar tissue formation, ventricular wall thinning, ventricular remodeling, and even cardiac failure (Xu et al., 2009). Recent studies have shown that transplantation of adipose-derived stem cells (ADSCs) in small animals can improve heart function after MI (Omar et al., 2019; Weng et al., 2019; Bao et al., 2020). ADSCs have been proposed to release angiogenic factors that protect cardiomyocytes from apoptosis and induce endogenous cardiomyocyte proliferation in addition to recruiting resident cardiac stem cells (Bai and Alt, 2010; Di Stefano et al., 2010; Lambert et al., 2019; Bao et al., 2020). Furthermore, transplanted cells can differentiate into cardiomyocytes and endothelial cells, which can form small vessels (Madonna and De Caterina, 2010).

However, there are two issues that must be addressed in clinical applications: leakage of the transplanted cells from the injection site and attack of the transplanted cells by the host. It has been reported that approximately 90% of cells can leak out of the injection site or emigrate into the circulation when delivered to the infarcted myocardium (Leor et al., 2000). Furthermore, there are studies demonstrating that allogeneic ADSCs transplanted into myocardial scar tissue survive for only a short period of time in the recipient heart due to immunorejection (Lee et al., 2015). Therefore, these main problems of cell loss and immuno-rejection must be solved immediately.

Tissue engineering presents great potential for providing solutions to the issues mentioned above, as it involves the use of living cells and biomaterial scaffolds to design and develop biological substitutes for tissue replacements. Several different biocompatible biomaterials have been used in the injectable cardiac tissue engineering. Biocompatible biomaterials used as scaffolds have included fibrin, chitosan (Lu et al., 2009), alginate (Naghizadeh et al., 2018), and self-assembled peptides (Lu and Wang, 2018). In our previous work (Lu et al., 2009), coinjection of embryonic stem cells with temperature-responsive chitosan hydrogel was shown to improve the function of the infarcted heart. Unfortunately, ethical issues have limited the experimental and clinical application of embryonic stem cells. Autologous ADSCs cells appear to be the optimal choice as living cells due to their immunoprivilege. Autologous cells allow complete avoidance of immunorejection, and ADSCs can be obtained easily from subcutaneous fat via minimally invasive procedures.

Recent clinical developments in tissue bioengineering have applications in acute cardiac ischemia and infarction and include the use of stem cells (such as ADSCs) combined with injectable scaffold material, such as Matrigel. Therefore, this study aimed to evaluate the effects of ADSCs combined with the Matrigel scaffold on cardiac morphology in a rat model of acute myocardial infarction.

## 2. Materials and methods

### 2.1. Animals

**Figure 1:**
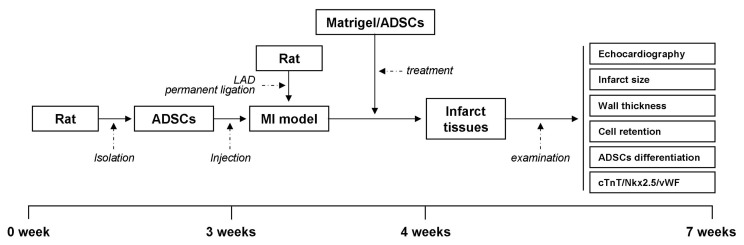
The schematic timeline of the surgical and experimental procedures of rats.

Rats were obtained from the Experimental Animal Center for the First Affiliated Hospital of Xinjiang Medical University (Xinjiang, China). All animal experiments were conducted at the Animal Center of Xinjiang Medical University. All animals were acclimatized under standard laboratory conditions (ventilated room, 25 ± 1 °C, 60 ± 5% humidity, 12 h light/dark cycle) and had free access to standard water and food. Efforts were made to limit the number of rats used and to minimize their suffering. All animals were anesthetized using the overdosage of pentobarbital prior to death. Subcutaneous adipose tissue from the inguinal groove was harvested from female Sprague–Dawley rats (SD) (80–120 g), which were then tagged. The wounds were later sutured and cared for. Coronary artery ligation was performed 3 weeks after this experiment. The schematic timeline of the surgical and experimental procedures of rats are shown as Figure 1.

All animal care, husbandry, and experimentation were in compliance with the ARRIVE guidelines (Percie du Sert et al., 2020). These experiments or tests were approved by the Animal Care and Use Committee of the First Affiliated Hospital of Xinjiang Medical University (Approval No. IACUC-20190225-48).

### 2.2. Isolation and culture of ADSCs

The isolation of ADSCs and the culture of ADSCs were carried out as described by a previously published study (Chu et al., 2019), with some modifications. Briefly, the adipose tissues were washed with 0.1 M phosphate-buffered solution (PBS, adjustment pH, 7.4, Gibco, NY, USA) for 3 times. The above tissues were then cut into slices and digested using 1 mg/mL type I collagenase (Cat. No. SCR103, Sigma-Aldrich, St. Louis, MO, USA) for 40 min at 37 °C using gentle agitation. Subsequently, the above cells were resuspended with the α-minima essential medium (α-MEM) (Cat. No. 21090055, Gibco Biotech., Grand Island, NY, USA) supplementing with 15% fetal bovine serum (FBS) (Cat. No. 10099, Gibco Biotech., Grand Island, NY, USA) and the antibiotics (Gibco Biotech., Grand Island, NY, USA), and filtered using a mesh filter (70 μM) (Cat. No. 352350, BD Biosciences, Grand Island, NY, USA), centrifuged for 6 min at a speed of 600 × *g*. The cells were then seeded on six-well plates (34.8 mm) (Cat. No. CLS3516-50EA, Corning Costar, Cambridge, MA, USA) and cultured under the circumstance of 5% CO_2_ at 37 °C. Obviously, a few spindle-shaped cells were discovered and observed in visible symmetric colonies obtained after 4 to 5 days. The cells were then subcultured with a density of 70%–80% confluence. ADSCs were harvested at the third passage through flow cytometry analysis as previously described (Chu et al., 2019). The cells were then fixed for 30 min in 2% paraformaldehyde in ice cold (Cat. No. P1116, SolarBio. Sci. Tech. Co. Ltd., Beijing, China) washed in cell staining buffer (Biolegend Inc., San Diego, CA, USA) and incubated with the following antibodies for 30 min: fluorescein isothiocyanate (FITC) conjugated anti-CD105 (Cat. No. 684102, Biolegend Inc., San Diego, CA, USA), FITC conjugated anti-CD90 (Cat. No. 561969, BD Biosciences Pharmingen, San Diego, CA, USA), phycoerythrin (PE) conjugated anti-CD34 (Cat. No. 826401, Santa Cruz Biotech., Dallas, Texas, USA), and FITC conjugated anti-CD45 (Cat. No. 560976, BD Biosciences Pharmingen, San Diego, CA, USA). FITC-conjugated secondary antibodies were subsequently added to detect the unlabeled primary antibodies. All analyses were performed using a BD flow cytometer (BD Bioscience, San Jose, CA, USA).

The ADSCs were labeled with the fluorescent cell-linker dyes PKH26 (Cat. No. PKH26PCL, 3822009000, Sigma-Aldrich, CA, USA) before injection, where 1 × 10^7^ cells were cocultured with PKH26 dyes at room temperature for 30 min.

### 2.3. Cell viability in Matrigel

The LIVE/DEAD staining method was performed with reference to a previously published study by Christman et al. (Christman et al., 2004). For these assays, 100 μL of Matrigel (temperature-responsive, Becton, Dickinson and Company, Franklin Lake, NJ, USA) was combined with 1 × 10^5^ ADSCs, followed by plating in four-well culture plates. The culture media were then added to the plates and changed every three days. LIVE/DEAD stain (Molecular Probes) was used to label living and dead cells after 24 h and 72 h (each culture time point was duplicated 5 times). A mixture of 5 μL of a 2 mM ethidium homodimer-1 solution and 1.25 μL of 4 mM calcein was produced in 5 mL of PBS, and each plate was incubated with 200 μL of the mixed solution for 30 min. To distinguish living cells from dead cells, we rinsed the ADSCs/Matrigel cultures with PBS and examined using a LEICA-DMIRB fluorescence microscope (Wetzlar, Germany). The obtained images were subsequently analyzed in Matlab to allow automated unbiased counting to distinguish living cells from dead cells.

### 2.4. Establishment of an autologous adipose-derived stem cell myocardial infarction model

Three weeks after harvesting subcutaneous adipose tissue, a model of myocardial infarction (MI) was induced by permanent ligation of the left anterior descending (LAD) coronary artery in female SD rats as previously described (Weng et al., 2019). Briefly, female SD rats were anesthetized with sodium pentobarbital (30 mg/kg, intraperitoneal), after which limb-lead electrocardiography was performed, followed by endotracheal intubation for ventilation and initiation of ventilation (Model: 683, Harvard Apparatus Rodent Ventilator, using room air, at a rate of 60 cycles/min, with a tidal volume of 1 mL/100 g body weight). The surgical approach involved a left lateral thoracotomy, pericardiectomy, and identification of the left anterior descending coronary artery (LAD) for permanent ligation. The left coronary artery was ligated with a 6-0 prolene suture placed 2–3 mm from its origin, between the conus of the pulmonary artery and the left atrium. After successful ligation, the infarct area of the left ventricle (LV) immediately paled and beat weakly. Electrocardiography showed typical MI waves (typical changes in the ST-segment). The rats were subsequently allowed to recover with care. Within the first 24 h following occlusion, the rats showed a mortality rate of 30%. The survived rats were fed and maintained freely accessing standard rat food (chow) and water.

### 2.5. Injection surgery

After one week, rats were randomly divided into 4 groups, with an inclusive criterion for the ejection fraction (EF) rate less than 40% according to echocardiography. Rats were reanesthetized, and their hearts were reexposed for injection surgery.

The rats operated by MI (98 remaining) were randomly divided into the following groups: PBS group, Matrigel group, PBS plus ADSCs group (PBS+ADSCs) and Matrigel plus ADSCs group (Matrigel+ADSCs). Female unoperated SD rats were used as the control group (n = 10). (1) Rats in the PBS group were injected using 100 μL of PBS (n = 21, among which 2 were used for Western blot analysis). (2) Rats in the Matrigel group were injected using 100 μL of Matrigel (n = 24, of which 8 were used for western blot analysis); (3) Rats in the PBS+ADSCs group were injected using the 2 × 10^6^ ADSCs in a PBS suspension (n = 26, among which 8 were used for Western blot analysis). (4) The ADSCs+Matrigel group was injected with 2 × 10^6^ ADSCs in a Matrigel suspension (n = 27, of which 8 were used for western blot analysis).

Rats were injected along the border of the infarct area at three different locations using a 28-gauge needle attached to the insulin syringe. Injections were performed at an angle to reduce the chance of injection into the lumen of the LV and were verified by a slight lightening in the color of the myocardium as the solutions entered the infarcted wall. Successful injections were defined as the formation of a bleb that covers the infarct zones. To reduce the overflow of ADSCs and Matrigel, we hold the needle in place for about 20 s after injection. The texts or experiments in the control group were performed with rats in the PBS group, while in the Matrigel group only injection was made with the PBS and Matrigel. The rats’ chests were then closed and allowed to recover with care.

### 2.6. Echocardiography

Transthoracic echocardiography was performed in rats after injections. This process was carried out in a blinded fashion using a 14.0 MHz probe (Sequoia 512; Acuson). Parasternal short-axis views with M mode were acquired at the ventricular base immediately distal to the mitral valve. The LV end-diastolic diameter (EDD) and the LV end-systolic diameter (ESD) were measured. LV fractional shortening (FS) and LV ejection fraction (EF) were calculated as follows: FS (%) = [(EDD−ESD)/EDD] ×100. EF (%) = [(EDD^3^−ESD^3^)/EDD^3^] × 100 (Carmona et al., 2017). An experienced researcher blinded to the rat groups performed all procedures and analyses. Rats were euthanized after functional measurements by an overdose of sodium pentobarbital, in accordance with the guidelines listed above.

### 2.7. Histology and immunohistochemical staining

Four weeks after the operation, the rats were euthanized after functional measurements through an overdose of sodium pentobarbital. Subsequently, the hearts of the rats were isolated and fixed with 4% paraformaldehyde (Cat. No. P1110, SolarBio. Sci. Tech. Co. Ltd., Beijing, China). The hearts of the rats were then also cut into 2 transverse slices through the infarct area and embedded in paraffin for the preparation of paraffin-embedded sections (4 μm). To measure cell retention within the myocardium 24 h after implantation, the area covered by PKH26-labeled ADSCs was traced using a fluorescent microscope (Model: BX51, Olympus, Tokyo, Japan). The ratio of the area covered by PKH26-labeled cells to the total infarct area in the sections was measured using RS Image Pro version 4.5 (Media Cybernetics, Inc. Bethesda, MD, USA). Cell retention was reported as the percentage of the area labeled with PKH26 within the infarct area.

To determine the size of the infarct and the thickness of the left ventricular wall, five sections were prepared at 5 different transverse levels at the tissue necrosis site, encompassing the entire infarct area. Sections (4 μm) were stained with Masson’s trichrome (Cat. No. 100485, Sigma, St. Louis, St. Louis, MO, USA) and evaluated using computer-assisted planimetry. The size of the infarct was quantified as the percentage of the total endocardial circumference of the left ventricle occupied by the infarct endocardial circumference (Qiao et al., 2019). Scar thickness was determined based on an average of 3 equidistant measurements in each section.

The number for arterioles was verified by immunohistochemical staining of tissue sections, using the vWF antibody. A total of five sections (with a thickness of 4 μm) including all infarcted areas (from base to apex with intervals of about 1 mm) were selected and collected from the heart of each rat in all 4 groups. The arterioles in each section of heart tissues were quantified with the following listed criteria: (1) The positive for vessel-endothelium labels. (2) Locate within the areas of the infarct scar. (3) Demonstrating a visible lumen. (4) Illustrating a diameter ranging from 10 mm to 100 mm (Qiao et al., 2019). The number of arterioles was defined as accounts of arterioles per mm^2^. For the immunofluorescence stain analysis, heart tissues were isolated and embedded and frozen in liquid nitrogen. Four-micrometer thick tissue sections were then incubated with primary antibodies (cardiac troponin T (cTnT), Sigma, 1:800, St. Louis, MO, USA; von Willebrand factor (vWF), Zymed, 1:200, San Diego, California, USA; NK2 transcription factor related locus 5 (Nkx2.5), Sigma, 1:200, St. Louis, MO, USA) at 4 °C overnight. Subsequently, the sections were incubated with FITC-conjugated IgG (Sigma, 1:100, St. Louis, MO, USA) for 1 h at room temperature, before being examined by confocal microscopy (Model: FV1000S, Olympus, Tokyo, Japan).

### 2.8. Western blot

To evaluate ADSCs differentiation in the infarct area, heart tissues were obtained from the infarct area in individual rats, and comparisons were made between the four groups based on Western blot analysis. Protein extracts (40 μg) were resolved on 10%–12% SDS polyacrylamide gels and transferred to polyvinylidene fluoride membranes. The membranes were then blocked in 5% nonfat dry milk in Tris-buffered saline containing 0.05% Tween-20 (TBST) for 1 h at room temperature. After blocking for 120 min, immunodetection was performed, first against cTNT (Cat. No. 15513-1-AP, 1: 500, Proteintech., Rosemont, IL, USA), Nkx2.5 (Cat. No. ab97355, 1: 500, Abcam Biotech., Cambridge, MA, USA) and vWF (Cat. No. ab174290, 1: 500, Abcam Biotech.) and then against the total level of each protein and GAPDH (Cat. No. 60004-1-Ig, 1: 500, Proteintech.). After incubation with primary antibodies overnight, cells were washed and incubated with horse radish peroxidase (HRP) labeled goat antirabbit IgG (Cat. No. A0208, Beyotime Biotech., Shanghai, China). A Western blot detection kit (Cell Signaling Technology, Danvers, MA, USA) was used to visualize the resultant bands. The photon intensity of tissue samples was normalized to control samples in arbitrary densitometric units.

### 2.9. Statistical analysis

The data presented herein were based on the multiple experiments or measurements, with STD deviation. Tukey’s multiple comparison tests validated ANOVA was applied to determine the possible significant differences in infarct size, cardiac functions, wall thickness, and the vessel density between groups. Statistical analyses were performed with SPSS 17.0 statistical software (SPSS, Inc., Chicago, IL, USA). The *p*-value less than 0.05 was defined as statistically significant.

## 3. Results

### 3.1. Dead rats in an experimental process

A total of 150 rats were used in this study. A total of 17 rats died after liposuction surgery. A total of 16 rats died during or immediately after infarct surgery and 19 rats died during injection surgery (PBS-only group, PBS+ADSCs group, Matrigel-only group, ADSCs+Matrigel group). After injection surgery, 100% survival was observed in all groups.

### 3.2. Characterization of adipose-derived stem cells

**Figure 2:**
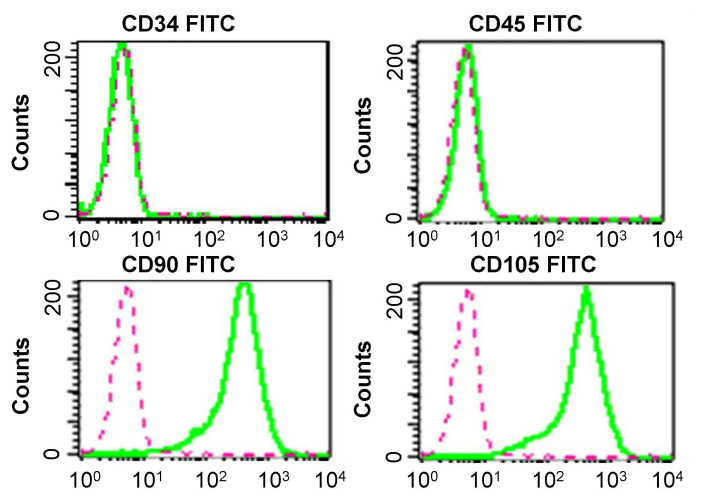
Identification of adipose-derived stem cells (ADSCs) with flow cytometry analysis. Most of ADSCs cultured expressing the CD105 and CD90 molecule, while negative for the CD34 and CD45 molecule.

To confirm the immunophenotype of the cultured cells, flow cytometry was performed with a FACscan argon laser cytometer after approximately three to four passages. Most cells expressed CD105 and CD90. One the contrary, the majority of adherent cells were negative for CD34 and CD45, as indicated in Figure 2.

### 3.3. Cell viability in Matrigel

**Figure 3:**
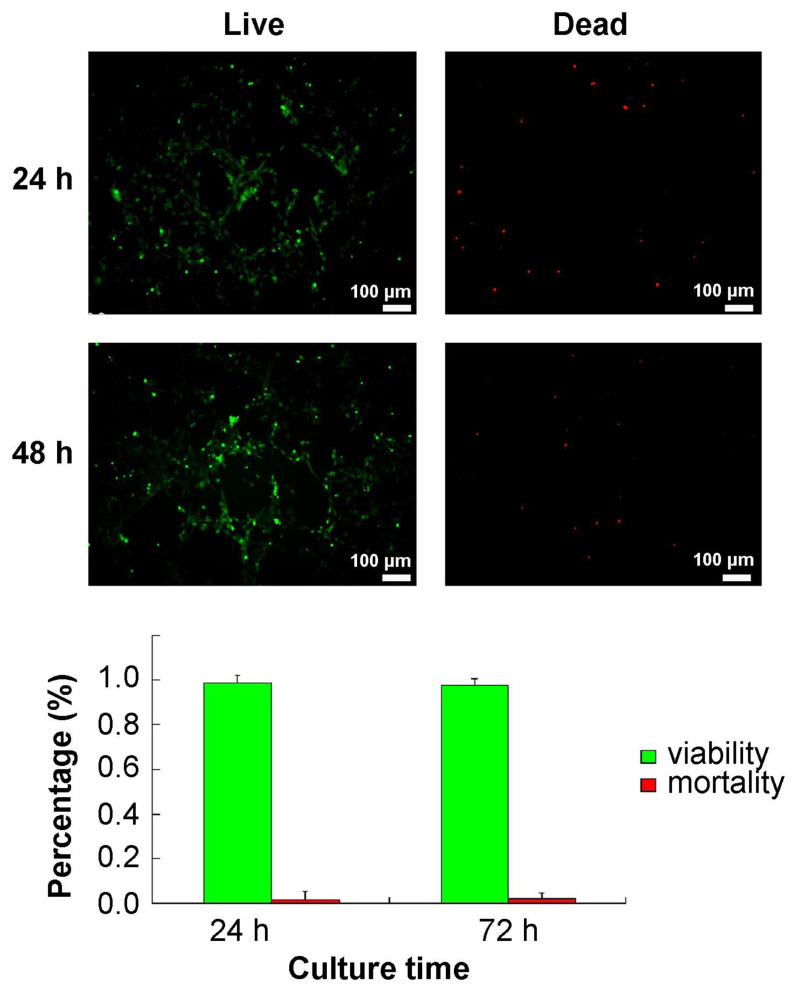
Evaluation of the viability of adipose-derived stem cells (ADSCs) in Matrigel. A. Live/dead staining for ADSCs 24 h and 72 h after seeding in Matrigel. Live cells were stained green, while dead cells were stained red. The bars = 100 μm. B. The results showed that ADSCs seeded and cultured in Matrigel maintained more than 90% cell viability at 24 h and 72 h.

Live/Dead staining demonstrated that ADSCs survived and proliferated well in Matrigel (Figure 3A). This assay showed a normal morphology, and ADSCs maintaining >90% viability at 24 h and 72 h (Figure 3B). Cell death was rarely observed due to the Matrigel.

### 3.4. Degradation of the Matrigel in the infarct heart

**Figure 4:**
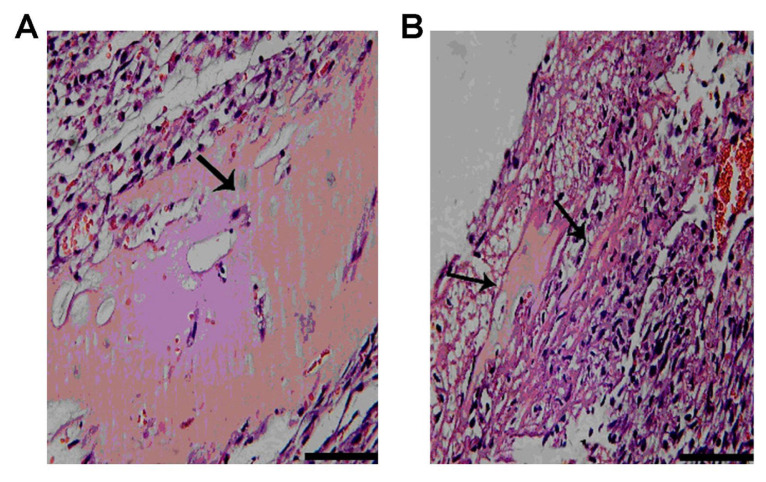
Observation of Matrigel degradation in the infarct heart. A. Hematoxylin and eosin staining showed that there was a large amount of matrigel in the infarct area after 24 h of transplantation. B. There was a small amount of matrigel in the infarct area after 4 weeks of transplantation. Bars = 100 mm.

HE staining showed a considerable amount of Matrigel (pink) in the infarct area 24 h after transplantation, as shown in Figure 4A, while there was little Matrigel in the infarct area 4 weeks after transplantation (Figure 4B).

### 3.5. Cell retention

**Figure 5:**
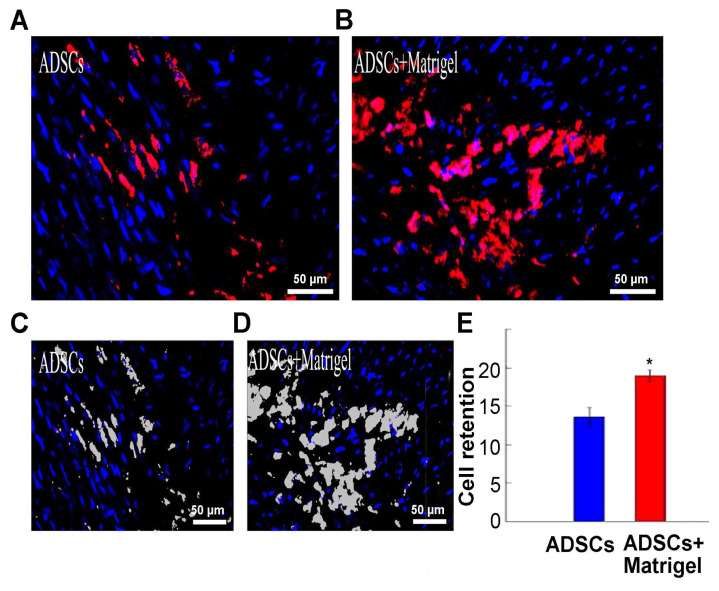
Analysis of PKH26-labeled adipose-derived stem cells (ADSCs) 24 h after the injection. A. In the ADSCs group: PKH26-labeled ADSCs scattered in the infarct area. In the Matrigel+ADSCs group: PKH26-labeled ADSCs accumulated in the infarct area. The red fluorescence illustrated ADSCs cells labeled with PKH26 and the blue fluorescence illustrated the cell nucleus. The gray areas marked by the RS image Pro. The two lower images showed the graft size of the upper images separately. B. The diagram shows the statistical results. ^*^p < 0.05 compared to the ADSCs group. Bars = 50 μm.

At 24 h after injection, ADSCs injected in PBS covered 13.58 ± 1.23% of the infarct, while ADSCs injected in the Matrigel covered 18.99 ± 0.07% (Figure 5A). The gray areas marked by RS image PRO in Figure 5A (lower images) represent the graft sizes of the upper images. The differences between the ADSCs group and the ADSCs+Matrigel group were significant (Figure 5B, p < 0.01).

### 3.6. Cardiac function evaluation (echocardiography)

**Figure 6:**
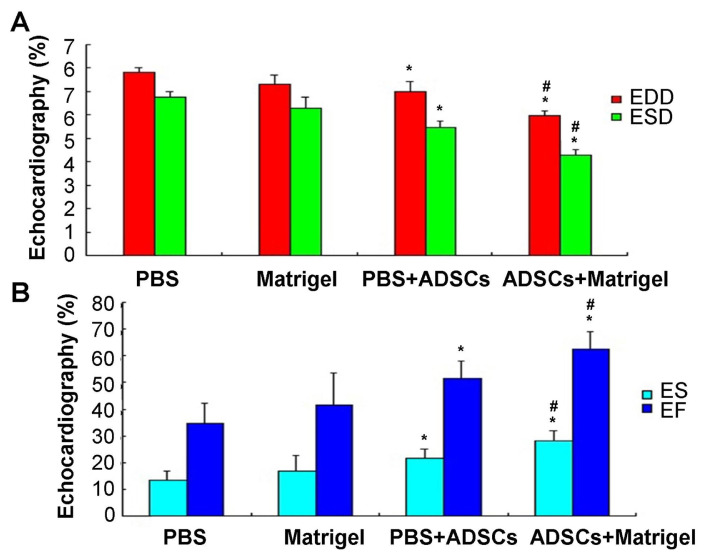
Diagram showing the statistical results of echocardiography. A. Echocardiography findings for left ventricular end-diastolic diameter (LVEDD) and left ventricular end-systolic diameter (LVESD). B. Echocardiographic findings for left ventricular ejection fraction (LVEF) and left ventricular fractional shortening (LVFS). ^*^p < 0.05 compared to the PBS group and the Matrigel group, ^#^p < 0.05 compared to the PBS+ADSCs.

The EDD, ESD, FS, and EF values were evaluated and analyzed as shown in Figures 6A and 6B. The PBS+ADSCs group (6.97 ± 0.19 mm) and the ADSCs+Matrigel group (5.97 ± 0.15 mm) exhibited marked improvements in LVEDD, LVESD (Figure 6A), LVFS, and LVEF (Figure 6B) compared to those of the PBS group (7.81 ± 0.21 mm) and the Matrigel group (7.68 ± 0.30 mm) 4 weeks after injection operation (p < 0.05). Compared to the PBS group (6.83 ± 0.07 mm) and the Matrigel group (6.77 ± 0.05 mm), the LVESD of the PBS+ADSCs group (5.03 ± 0.08 mm) and the ADSCs+Matrigel group (4.21 ± 0.05mm) also improved significantly (Figure 6A, p < 0.05). Consistently, compared to the PBS group (15.80%±1.00%) and the Matrigel group (15.60% ± 1.00%), LVFS of the PBS+ADSCs group (25.60% ± 2.00%) and the ADSCs+Matrigel group (35.50% ± 2.00%) also significantly increased (Figure 6B, p < 0.05). LVEF was also significantly improved in the PBS+ADSCs group (56.64% ± 5.0%) and the ADSCs+Matrigel group (62.74% ± 3.0%), compared to the PBS group (39.70% ± 4.00%) and the Matrigel group (Figure 6B, 40.73% ± 4.00%). According to the above findings, rats treated with ADSCs+Matrigel exhibited the most improved heart function compared to those of the PBS+ADSCs group (Figures 6A and 6B, p < 0.05). Data also showed that compared to the PBS-only group, the Matrigel group presented improvements in LVESD, LVEDD, LVFS, and LVEF, however, without significant differences between the PBS group and the Matrigel group (Figures 6A and 6B, p > 0.05).

### 3.7. Infarct size

**Figure 7:**
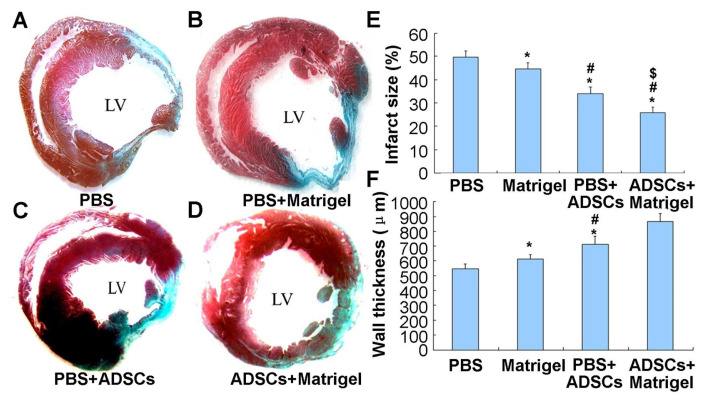
Evaluation for the size and thickness of the infarct wall. Masson trichrome staining of representative sections showed an infarct wall and an infarct size of the four groups. A. The infarct area in the PBS group, the Matrigel group, the PBS+ADSCs group, the ADSCs+Matrigel group. B. Diagram showing the statistical results of infarct size. C. Diagram showing the statistical results of the thickness of the infarct wall. ^*^p < 0.05 compared to the PBS group. ^#^p < 0.05 compared to the Matrigel group. ^$^p < 0.05 compared to the PBS+ADSCs group. ADSCs: adipose-derived stem cells.

The Masson trichrome findings showed that fibrous tissues appeared in samples of heart tissues. The infarct myocardium substituted/replaced with fibroblasts and/or collagen appeared to be blue, while the viable myocardium appeared to be red. The size of the infract was defined as the following: the ratio of the infarct endocardial circumference versus (*vs.)* the total endocardial circumference for the left ventricle, according to the descriptions above. Compared to the PBS group (Figure 7A, 49.68 ± 2.69%), the infarct size in the Matrigel group (Figure 7A, 44.55 ± 2.62%), the PBS+ADSCs group (Figure 7A, 33.90 ± 3.11%) and the ADSCs+Matrigel group (Figure 7A, 25.86 ± 2.51%) was significantly reduced (p *<* 0.05). The infarct size was significantly reduced in the PBS+ADSCs group compared to the Matrigel group (p *<* 0.05). Rats administering ADSCs+Matrigel showed a more reduced infarct size compared to those of rats administering PBS+ADSCs (Figure 7B, p < 0.05).

### 3.8. Wall thickness

The thickness of the ventricular wall in the center of the myocardial infarction 4 weeks after implantation (Figure 7C) was 545.5 ± 34.53 μm in the PBS group, 613.3 ± 29.5 μm in the Matrigel group, 709.5 ± 54.6 μm in the PBS+ADSCs group, 866.12 ± 54.76 μm in the ADSCs+Matrigel group. Compared to the PBS group, the thickness measured in the Matrigel group, the PBS+ADSCs group, and the ADSCs+Matrigel group was remarkably higher (Figure 7C, p < 0.01). Cotransplantation for ADSCs and Matrigel showed the best effect in keeping the thickness of the ventricular wall compared to that of the ADSCs and Matrigel treatment group (Figure 7C, p < 0.01). No significant differences were observed between the Matrigel and ADSCs groups (Figure 7C, p > 0.05).

### 3.9. Differentiation for ADSCs of infarct hearts

**Figure 8:**
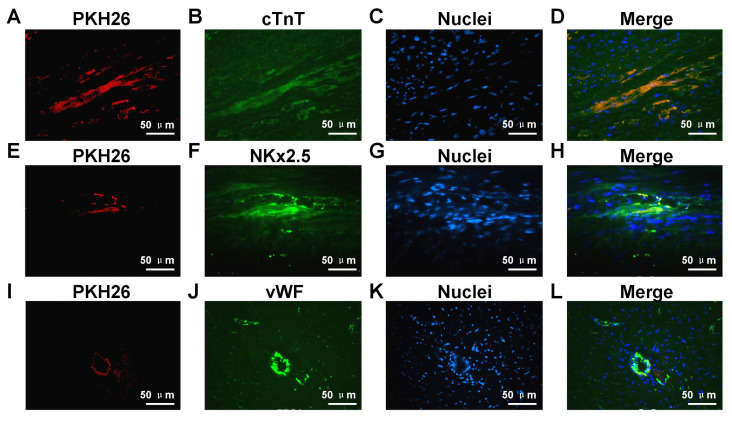
Determination of ADSCs differentiation in the infarct heart. Differentiation of coinjection ADSCs and Matrigel in the infarct heart 4 weeks after transplantation. Representative immunofluorescence images of PKH26 labeled cells immunostained with antibodies against cardiac troponin T (cTnT) (A–D), NK2-transcription factor-related locus-5 (Nkx2.5) (E–H) and von Willebrand factor antigen (vWF) (I–L). Bars = 50 μm.

The cells grafted into myocardial tissue sections were determined according to the appearance of PKH26. The present cTnT immunofluorescence staining findings along with the PKH26 label of ADSCs after 4 weeks of transplantation suggested the existence of PKH26-positive and cTnT-positive cells in infarcted areas, as shown in Figure 8A. The results presented in Figure 8B illustrated that colocalization for the Nkx2.5 molecule and the PKH26 molecule in the infarct zone, potentially showing the formation of neonatal cardiomyocytes. Furthermore, a small fraction of PKH26-positive ADSCs exhibited positive staining for vWF, showing that they might form endothelial cells, as shown in Figure 8C. The results indicate a role for ADSCs grafted in the formation of the neovasculature in myocardial infarction.

### 3.10. Formation of the neovasculature

**Figure 9:**
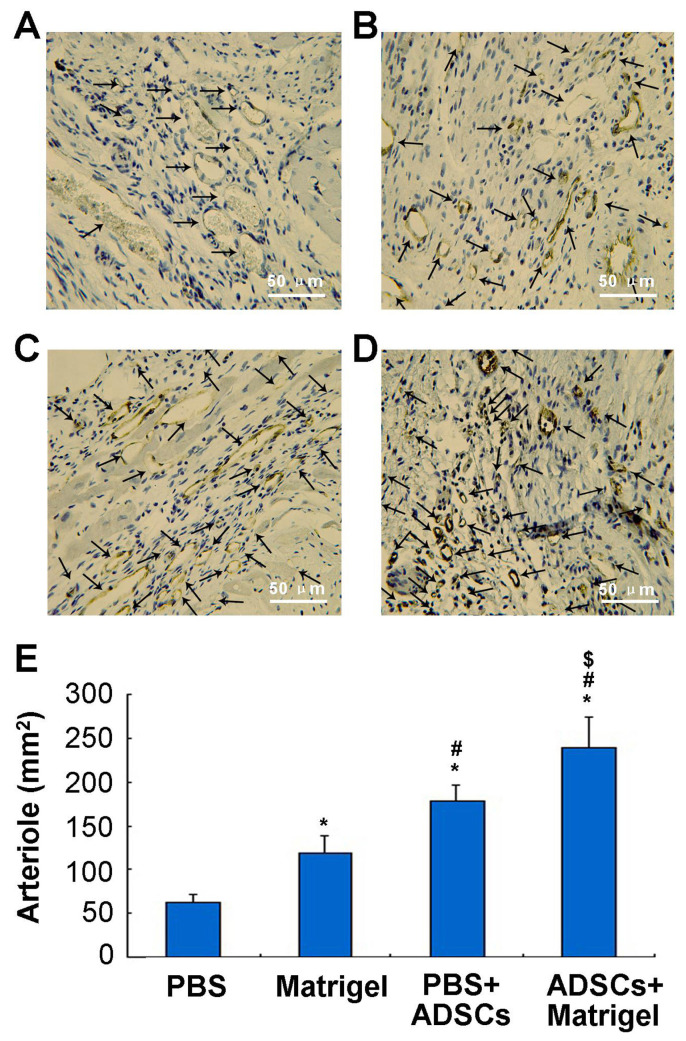
Evaluation of arteriole density in the infarct area 4 weeks after injection. Arteriole density (von Willebrand factor (vWF) stain) in the infarct area 4 weeks after injection. A. Arteriole density in the PBS group, Matrigel group, the PBS+ADSCs group, and the ADSCs+Matrigel group. B. Diagram showing the statistical results of arteriole density. ^*^p < 0.05 compared to the PBS group, ^#^p < 0.05 compared to the Matrigel group. ^$^p < 0.05 compared to the PBS+ADSCs group only. The black arrows showed the arterials. Bars = 50 μm. ADSCs: adipose-derived stem cells.

The potential for neovasculature formation was evaluated using immunohistochemical analysis with the vWF antibody (Figure 9A). Arterioles were counted to evaluate the neovascular effect in different treatments. Four weeks after implantation, the arteriole densities in the central area of myocardial infarction were as follows (Figure 9B): 63.06 ± 8.9/mm^2^, 118.2 ± 20/mm^2^, 179.4 ± 17/mm^2^, and 239 ± 16/mm^2^, in the PBS group, the Matrigel group, the PBS+ADSCs group, and the ADSCs+Matrigel group, respectively. The arteriole density of the scarred areas increased remarkably in the Matrigel group, the PBS+ADSCs group and the ADSCs+Matrigel group compared to the PBS group (Figure 9A) (p < 0.05). As shown in Figure 9B, the greatest improvement of arteriole density was discovered in the ADSCs+Matrigel group (p < 0.05). Meanwhile, an obvious difference was also discovered between the PBS+ADSCs and Matrigel group (Figure 9B, p < 0.05).

### 3.11. Western blot analysis

**Figure 10:**
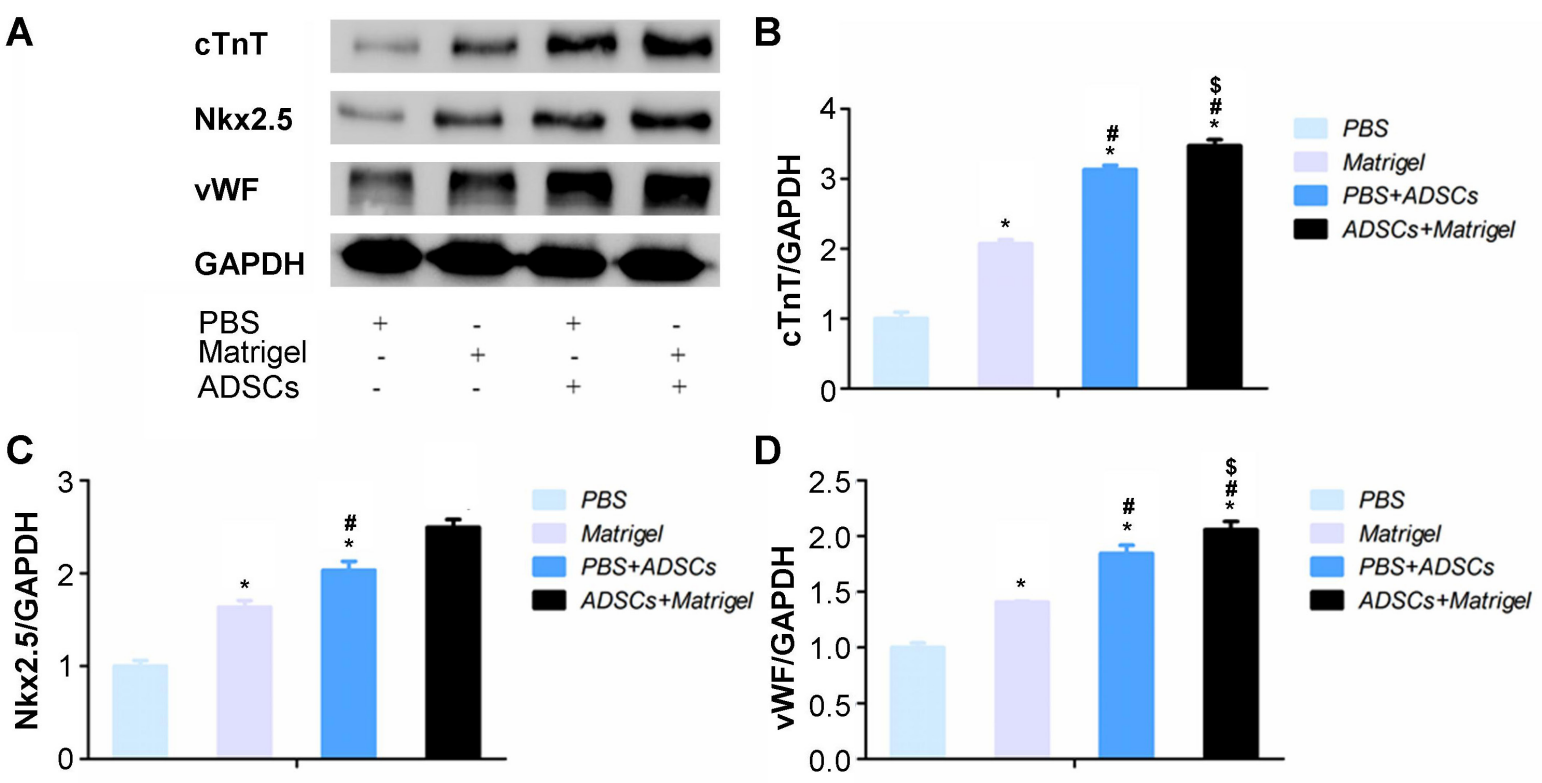
Verification of the expression of cardiac troponin T (cTnT), NK2-transcription factor related locus-5 (Nkx2.5) and von Willebrand factor antigen (vWF). A. Western blot images for the expression of cTnT, Nkx2.5 and vWF expression in the injured myocardium. B. Semiquantitative assay of cTnT expression by optical density value. C. Semiquantitative assay of Nkx2.5 expression by optical density value. D. Semiquantitative assay of vWF expression by optical density value. ^*^p < 0.05 compared with PBS group, ^#^p < 0.05 compared with Matrigel group, ^$^p < 0.05 compared with PBS+ADSCs group. ADSCs: adipose-derived stem cells.

Western blot identified that all treatment groups obviously exhibited increased levels of cTnT, Nkx2.5 and vWF protein compared to those of the PBS group (Figure 10A, p < 0.05). Cotransplantation with ADSCs group and the Matrigel and PBS+ADSCs group showed significantly increased expressions of the cTnT (Figure 10B), Nkx2.5 (Figure 10C) and vWF (Figure 10D) molecule compared to those of the Matrigel group (p < 0.05). Comparing to the PBS+ADSCs group, the expressions of molecules in the ADSCs+Matrigel group were predominantly higher (Figures 10A–10D, p < 0.05).

## 4. Discussion

In this study, we cotransplanted ADSCs and Matrigel in rat model of myocardial infarction to improve cardiac morphology and cardiac function. The findings showed that the ADSCs combining Matrigel exhibited an obvious improvement in heart function (LVESD, LVEDD, LVFS, LVEF) and a significant reduction in the size of the infarct. Cotransplantation of ADSCs and Matrigel showed a significant effect on maintaining the thickness of the ventricular wall. Engrafted ADSCs played a role in the formation of neovasculatures in myocardial infarction. Cotransplantation of ADSCs and Matrigel triggered remarkable enhancement of arteriole density and displayed significantly higher levels of cTnT, Nkx2.5, and vWF. Therefore, our study demonstrated that cotransplanting ADSCs with Matrigel resulted in an improvement in cardiac morphology and cardiac function in the rat model of myocardial infarction.

Despite progress in the medical sciences, coronary artery disease remains a major cause of death. Increasing evidence suggests that cell transplantation in an infarct myocardium might improve left heart function (Díaz-Herráez et al., 2017; Gálvez-Montón et al., 2017; Ma et al., 2017; Abd Emami et al., 2018; Şovrea et al., 2019). Bone marrow stem cells have been used in clinical applications in some cases (Han et al., 2019; Storti et al., 2019; Chu et al., 2020). In recent years, similar results have been obtained in experiments where ADSCs transplantation was performed after MI, resulting in significantly improved cardiac functions (Omar et al., 2019; Weng et al., 2019; Bao et al., 2020), showing better results compared to the use of bone marrow mononuclear cells (Karpov et al., 2013). Bagno et al. (Bagno et al., 2012) investigated the therapeutic efficacy of ADSCs in the myocardial infarction rat model and found that ADSCs could improve cardiac function; however, the injectable scaffold has not been used.

Actually, many three-dimensional polymer scaffolds, such as Matrigel, fibrin, collagen I, and hydrogels, have been used to support the matrices to deliver cells into infarcted cardiac muscle (Huang et al., 2005; Li and Guan, 2011). In this study, we selected the Matrigel as the injectable three-dimensional polymer scaffold and combined with ADSCs for the in myocardial infarction. Meanwhile, Ou et al. (Ou et al., 2011) also evaluated the therapeutic effects of intracardiac Matrigel injection in rat model of myocardial infarction, but they did not combine Matrigel with stem cells directly. Moreover, Kofidis et al. (Kofidis et al., 2005) generated injectable bioartificial tissue and proved that it could facilitate less invasive, large-scale tissue restoration in the beating heart after myocardial injury. A recently published systematic review and meta-analysis of preclinical studies on bioactive scaffolds used to evaluate stem cell therapies concluded that the use of cardiac progenitor cells in these models had the most significant effect on fractional shortening (Khan et al., 2021). Therefore, the greatest improvements in the cardiac ejection fraction were found when stem cell-embedded scaffolds were used. The present study demonstrated that transplantation of autologous ADSCs with Matrigel improved cell retention and survival. Heart function, infarct size, wall thickness, microvessel density within infarct areas 4 weeks after implantation were better in the ADSCs+Matrigel group compared to other groups. The underlying mechanisms must be explored in more depth.

In this study, the mechanical support and the internal structural were provided in the damaged area with the Matrigel. It has been reported that injection of biomaterials alone into infarct areas can limit the LV remodeling process and can also improve LV function. Differences in infarct size were observed in each group based on thickening of the ventricular wall (Chen et al., 2015; Chen et al., 2018; Lu and Wang, 2018). Our findings showed that, compared to the PBS group, the Matrigel group showed significant differences in the thickening of the ventricular wall and the size of the infarct. A greater wall thickness and smaller infarct size induce more local force-generating units for growth across the infarct wall, which might derive directly from the Matrigel. A form study (Efraim et al., 2017) has shown that Matrigel can repair injured myocardium without geometric distortion. This finding suggested that the Matrigel could provide mechanical support after gelatification in vivo and that the Matrigel matrix may provide mechanical support for the left ventricle by increasing the stiffness. After transplantation, the scaffold could temporarily provide biomechanical support for ADSCs to produce their own extracellular matrix.

In this study, we hypothesized that as a temporary semirigid scaffold, the complex protein mixture of Matrigel may provide the temporary extracellular matrix to increase cell survival (Feaster et al., 2015). Matrigel contains a number of basement membrane proteins, including fibroblast growth factor and transforming growth factor-β (TGF-β) and is often used to create a bioactive environment with multiple growth factors (Zhou et al., 2013). This composition can induce ADSCs angiogenesis and ADSCs proliferation in ischemic areas, which could help improve ADSCs survival (Grabowska et al., 2019). A former study (Huang et al., 2005) reported that the Matrigel as an injectable biopolymer could form the three-dimensional polymer scaffold and modulate the angiogenesis; however, this study has not combined ADSCs. It has been observed that ADSCs inoculation in the Matrigel could be responsible for its prolonged survival and improvement of cardiac function (Sheu et al., 2019). Our data showed that cell retention at 24 h was significantly greater when Matrigel was used compared to the PBS control. The percentage of 24 h of cell retention observed in the Matrigel group was 18.99 ± 0.07%, whereas that in the PBS group was 13.58 ± 1.23%. Furthermore, the data revealed that the arteriole density of the scar areas was predominantly increased in the Matrigel group compared to the PBS group. An adequate blood supply and high cell retention could ensure survival of ADSCs. Matrigel might guarantee other effects in the infarct area, such as differentiation and synchronization of beating with the host myocardium, which directly improves contractility and heart function. The echocardiography data confirmed our hypothesis: compared to the PBS treatment group, the improvement of LVESD, LVEDD, LVFS, and LVEF in the Matrigel group followed a trend of decline, although it was not statistically significant.

Huang et al. (2005) showed that fibrin, collagen I, and Matrigel as injectable biopolymers significantly enhanced myofibroblast infiltration into the infarcted area, with potential clinical benefits for repair postmyocardial infarction. We also speculated that direct differentiation of ADSCs was also one of the significant factors in improving cardiac function. ADSCs have been shown to be similar to bone marrow MSCs (bMSCs) in terms of, for example, mulitipotency and paracrine effects (Kellner et al., 2015; Weng et al., 2019). A previous study [31] has shown that intracardiac injection of Matrigel can induce stem cell recruitment and improve cardiac function in the rat myocardial infarction region. Compared with bMSCs, ADSCs have the advantages of convenient sampling, abundant source, simple isolation and culture method, fast proliferation rate, and strong amplification ability, especially suitable for autologous sampling application. Immunofluorescence of ADSCs labeled with PKH26 for cTnT 4 weeks after transplantation showed that both PKH26-positive and cTnT-positive cells existed in infarcted areas. Some PKH26-positive cells were also found to be vWF-positive, suggesting that they formed small blood vessels within the infarct zone. The arteriole density of the scar areas was obviously enhanced in the PBS+ADSCs group compared to that of the PBS group and the Matrigel group. Furthermore, our experimental data showed that compared to the PBS group and the Matrigel group, the PBS+ADSCs treatment presented significant differences in that the area of the myocardial infarction was reduced; the thickness of the ventricular wall in the area of the myocardial infarct was increased; and cardiac function was improved in our model of rat myocardial infarction. Therefore, Matrigel+ADSCs treatment was most effective among the treatments examined. We suggest that the synergy between Matrigel and ADSCs could improve heart function. Schwach et al. (2020) reported that the presence of Matrigel provides a basis for cardiomyocytes to extend cell-cell contacts. We subsequently performed further verification. The Western blot results again demonstrated that t Matrigel+ADSCs treatment was the most effective treatment. The cTNT, Nkx2.5, and vWF markers were detected in injured heart tissue. These markers were expressed at higher levels in the Matrigel+ADSCs group compared to other groups.

In addition, we cannot ignore the possibility of immunorejection. Although some studies have shown that ADSCs might suppress immunoreaction (Chow et al., 2017; Bobyleva et al., 2019), Jeffrey M proposed that transplanted allogeneic ADSCs would not evoke a strong immune response and that subsequent rejection requires independent and comprehensive testing (Konno et al., 2013). These controversies demonstrate that no ultimate conclusion has been reached regarding immunoreaction in ADSCs transplantation. In the present study, the encapsulation of autologous ADSCs within Matrigel was investigated for the treatment of MI, which could allow complete avoidance of immunorejection.

This study aimed to test the usage of Matrigel to provide a three-dimensional extracellular matrix for adipose-derived stem cells in the ischemic myocardium. The use of Matrigel could prevent the leakage of adipose-derived stem cells out of the injection site and prevent the host from attacking the transplanted cells. The results of our study highlight the potential benefit of Matrigel as an injectable scaffold or cell delivery vehicle to the infarct zone after infarction.

There are also some limitations to this study. First, the beneficial effects for LV remodeling and cardiac function were only assessed 4 weeks after injection. However, the functional changes and the architectural changes should be determined after a relatively longer time. Second, the specific mechanism by which Matrigel injection improved cardiac function is not entirely clear. Third, there is even no direct evidence that Matrigel could increase cell proliferation or neovasculature formation. The following studies should be conducted to investigate the above issues.

## 5. Conclusion

Using the myocardial infarction rat, this study showed that cotransplantation with ADSCs and the Matrigel could remarkably increase the number of arterioles, reduce the infarct size, alleviate the ventricular remodeling process, and improve/promote cardiac functions. Furthermore, cotransplantation of ADSCs and Matrigel demonstrated a higher efficacy compared to that of applying one type of treatment (ADSCs or Matrigel treatment). In summary, the findings of this preliminary study in a rat model of acute myocardial infarction demonstrated that cotransplantation of ADSCs and Matrigel resulted in improved cardiac morphology and cardiac function.
